# Genome-wide RNAi screen for nuclear actin reveals a network of cofilin regulators

**DOI:** 10.1242/jcs.169441

**Published:** 2015-07-01

**Authors:** Joseph Dopie, Eeva K. Rajakylä, Merja S. Joensuu, Guillaume Huet, Evelina Ferrantelli, Tiao Xie, Harri Jäälinoja, Eija Jokitalo, Maria K. Vartiainen

**Affiliations:** 1 Program in Cell and Molecular Biology, Institute of Biotechnology, University of Helsinki, 00014 Helsinki, Finland; 2 Image and Data Analysis Core (IDAC), Harvard Medical School, Boston, MA 02115, USA; 3 Light Microscopy Unit, Institute of Biotechnology, University of Helsinki, 00014 Helsinki, Finland; 4 Electron Microscopy Unit, Institute of Biotechnology, University of Helsinki, Helsinki, Finland

**Keywords:** Actin, Nucleus, Cofilin, RNA interference

## Abstract

Nuclear actin plays an important role in many processes that regulate gene expression. Cytoplasmic actin dynamics are tightly controlled by numerous actin-binding proteins, but regulation of nuclear actin has remained unclear. Here, we performed a genome-wide RNA interference (RNAi) screen in *Drosophila* cells to identify proteins that influence either nuclear polymerization or import of actin. We validate 19 factors as specific hits, and show that Chinmo (known as Bach2 in mammals), SNF4Aγ (Prkag1 in mammals) and Rab18 play a role in nuclear localization of actin in both fly and mammalian cells. We identify several new regulators of cofilin activity, and characterize modulators of both cofilin kinases and phosphatase. For example, Chinmo/Bach2, which regulates nuclear actin levels also *in vivo*, maintains active cofilin by repressing the expression of the kinase Cdi (Tesk in mammals). Finally, we show that Nup98 and lamin are candidates for regulating nuclear actin polymerization. Our screen therefore reveals new aspects of actin regulation and links nuclear actin to many cellular processes.

## Abstract (precis)

**Summary:** A genome-wide RNAi screen is used to identify several conserved regulators of nuclear actin import and polymerization, and thus links control of nuclear actin to many cellular processes.

## INTRODUCTION

Actin is a multifunctional protein in both the cytoplasm and the nucleus. In the cytoplasm, the dynamic regulation of actin polymerization into filaments and depolymerization into monomers, which is mediated by numerous actin-binding proteins (ABPs), is the key for its many functions during, for example, cell motility, intracellular transport and cell shape maintenance (Pollard and Cooper, 2009).

In the nucleus, actin is generally linked with protein complexes that are involved in all stages of gene expression (Grosse and Vartiainen, 2013). Actin specifically regulates the activity and localization of megakaryocytic acute leukemia protein MAL (also known as MKL1), a transcriptional coactivator of serum response factor (SRF) (Vartiainen et al., 2007). In addition, actin associates with many chromatin remodeling complexes (Zhao et al., 1998; Farrants, 2008; Kapoor et al., 2013) and has been shown to influence the function of all three RNA polymerases (Fomproix and Percipalle, 2004; Hofmann et al., 2004; Hu et al., 2004). Actin also interacts with the heterogeneous nuclear ribonucleoprotein U, and this association aids the recruitment of the histone acetylransferase PCAF to actively transcribing genes (Kukalev et al., 2005). The polymerization status of nuclear actin under physiological conditions is still debatable, although mounting evidence suggests that various forms of actin operate in the nucleus and might account for the diverse functions of actin in this compartment. For example, nuclear actin polymers have been shown to mediate de-repression of Toll-like receptor response genes through the actin filament-binding protein coronin 2A, a component of the nuclear receptor co-repressor (NCoR) complex (Huang et al., 2011). Similarly, nuclear actin polymerization has been linked to the activation of occluded pluripotent genes during somatic nuclear reprograming in the *Xenopus laevis* oocyte (Miyamoto et al., 2011). Recently, formin-regulated nuclear actin polymerization has been visualized for the first time and shown to regulate gene expression through the MAL–SRF pathway (Baarlink et al., 2013). By contrast, actin is kept monomeric at least in the yeast INO80 chromatin remodeling complex (Kapoor et al., 2013), and earlier studies have shown that polymeric actin associates with the Brm-associated factor (BAF) chromatin remodeling complex in a phosphatidylinositol-dependent manner (Rando et al., 2002). A recent report that utilized fluorescent probes based on known actin-binding domains showed that actin monomers are present in nuclear speckles, whereas actin polymers appeared to exclusively concentrate in interchromatin spaces (Belin et al., 2013). Despite the fact that numerous actin regulators are present in the nucleus (Rajakyla and Vartiainen, 2014), the mechanisms and signaling pathways that control nuclear actin polymerization are still unclear.

Although the detailed mechanisms through which actin regulates gene expression processes are lacking, the above findings strongly suggest that actin is an important protein in the nucleus. Indeed, decreased nuclear actin levels do not support maximal transcription in cells (Dopie et al., 2012). Moreover, low levels of nuclear actin seems to promote quiescence (Spencer et al., 2011), whereas increased nuclear actin has been linked to differentiation of HL60 cells towards macrophages (Xu et al., 2010). Nuclear actin levels might therefore play an important role in transcriptional regulation, and might even be used to elicit specific transcriptional programs, and thus cell fate decisions. This suggests that nucleo-cytoplasmic shuttling of actin must be tightly controlled. Actin appears to utilize active nuclear import (Dopie et al., 2012) and export (Stuven et al., 2003) mechanisms, although the size of actin (42 kDa) is close to the limit of passive diffusion. Nuclear export of actin is mediated by the transport factor exportin 6, and the small ABP profilin aids the interaction between actin and the exportin (Stuven et al., 2003). Another family of small ABPs, cofilins (represented by Tsr in *Drosophila*), plays a role in importin-9-dependent nuclear import of actin (Dopie et al., 2012). The signaling pathways regulating nuclear actin levels have remained largely unclear. A previous genome-wide screen in cultured *Drosophila* cells, followed by targeted screens in mouse and human cells, revealed that CG7597 and Hyx (known as Cdc73 and Cdc2l5, respectively, in mammals) are new regulators of nuclear actin (Rohn et al., 2011). Depletion of these factors by RNA interference (RNAi) caused the accumulation of actin in the nucleus, with a phenotype resembling exportin 6 depletion. In S2R+ cells this increased nuclear actin manifests as a phalloidin-stainable actin bar, and therefore these factors are candidates for acting either as nuclear export regulators of actin, or as negative regulators of nuclear actin polymerization. Importantly, the function of these proteins was conserved from flies to mammals (Rohn et al., 2011).

Here, we performed a genome-wide RNAi screen in cultured *Drosophila* cells to reveal new regulators of nuclear actin polymerization and proteins that influence nuclear actin levels by regulating its nuclear import. We confirm and validate the hits, and identify 19 specific regulators of nuclear actin, further demonstrating that a subset of these hits is also conserved in mammalian cells. Our results uncover new regulators of cofilin activity, which act at different levels to modify the phosphorylation status of this key actin regulator. We describe the transcriptional repressor Chinmo (Bach2 in mammals) as an *in vivo* regulator of nuclear actin levels, highlighting the importance of appropriate regulation of cofilin activity in this process.

## RESULTS

### Genome-wide screen in cultured *Drosophila* cells to identify new nuclear actin regulators

**Fig. 1.**
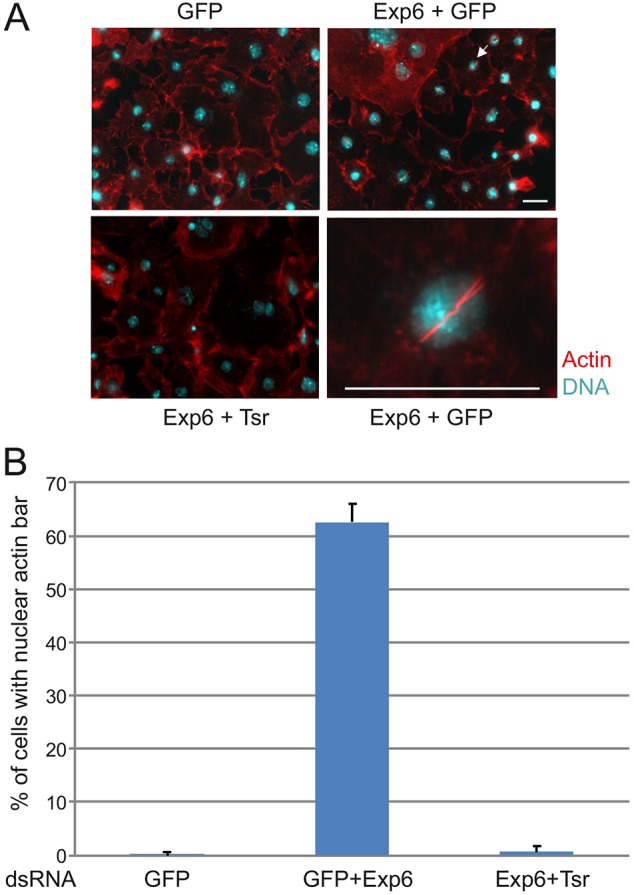
**Assay to identify novel nuclear actin regulating proteins.** (A) Representative images of *Drosophila* S2R+ cells stained with fluorescently labelled phalloidin (red) for actin filaments and DAPI (cyan) for DNA after cells were treated with dsRNAs against green fluorescent protein (GFP) or twinstar (Tsr) and exportin 6 (Exp6) as indicated. The arrow indicates the accumulation of a phalloidin-stainable nuclear actin bar in a exportin-6-depleted cell, which is shown at a higher magnification in the lower right panel. Scale bars: 10 µm. See also supplementary material Fig. S1A. (B) Quantification of the percentage of cells with the nuclear actin bar. Data is mean±s.d. of two independent experiments (200 cells per experiment).

One issue that has clearly hampered nuclear actin studies has been the difficulties associated with its visualization (Grosse and Vartiainen, 2013). The amounts of nuclear actin in most cells are very low compared to cytoplasmic actin, and therefore the nuclear signal is easily obscured by the strong cytoplasmic staining. Silencing of the nuclear export receptor for actin, exportin 6, results in nuclear accumulation of actin (Stuven et al., 2003). In cultured *Drosophila* S2R+ cells, this increased nuclear actin manifests as a phalloidin-stainable (i.e. filamentous) actin bar, and the number of cells containing this bar are easy to quantify by microscopy (Fig. 1A,B) (Dopie et al., 2012). Of note, these bars are exclusively found within the cell nucleus, as we showed in a previous confocal microscopy study (Dopie et al., 2012), and as demonstrated by electron microscopy (supplementary material Fig. S1A). Using this method to visualize nuclear actin, we recently identified Twinstar (Tsr), the *Drosophila* cofilin, as a regulator of nuclear localization of actin. Silencing Tsr suppresses the nuclear accumulation of actin, and therefore leads to disappearance of the nuclear actin bar caused by exportin 6 RNAi (Fig. 1A,B) (Dopie et al., 2012). In general, bar formation can be prevented either by inhibiting nuclear import of actin or impairing formation of the nuclear actin bar e.g. nuclear actin polymerization. This assay therefore represents an excellent tool to identify new proteins that regulate nuclear actin.

**Fig. 2.**
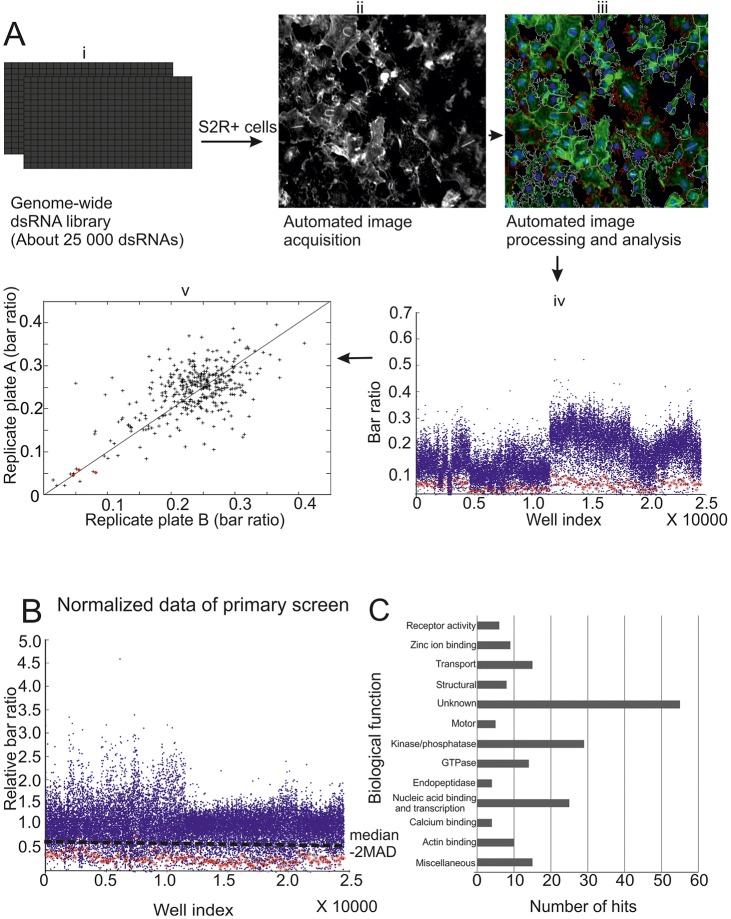
**Outline and summary of the genome-wide screen to identify regulators of nuclear actin.** (A) A scheme showing the workflow of the primary genome-wide RNAi screen in cultured *Drosophila* S2R+ cells. Cells were seeded in duplicate pre-aliquoted 384 wells containing the dsRNA library (i). Images were acquired after 5 days of RNAi (ii). Images were processed and analyzed (iii) as outlined in supplementary material Fig. S2. (iv) A scatter plot of bar ratios of the raw data from the whole primary screen, with blue dots representing individual data points and red dots representing the data from Tsr-dsRNA-treated wells (positive controls). (v) Scatter plot of raw data showing correlation between the bar ratio per well of representative replicate plates, with red points indicating bar ratio in Tsr-dsRNA-treated wells (positive control) and black indicating the rest of data points in the replicate plates. (B) Normalized data of the whole primary screen. The black dotted line indicates the approximate cut-off for hit selection using the 2 median absolute deviation (–2MAD) (see Materials and Methods). Blue dots represent individual data points and red dots the data from Tsr-dsRNA-treated wells (positive controls). (C) Predicted biological functions of candidate hits (wells of interest) from the primary screen. A list of these factors is found in supplementary material Table S1.

To identify all genes whose depletion prevents the bar formation in the absence of exportin 6, we performed a genome-wide RNAi screen in cultured *Drosophila* S2R+ cells. In brief (for details see Materials and Methods), cells were seeded in 384-well plates containing both the *Drosophila* RNAi Screening Center (DRSC) double-stranded RNA (dsRNA) library 2.0 targeting 13,900 *Drosophila* genes and the dsRNA targeting exportin 6. DsRNA against Tsr was used as a positive control. After 5 days of RNAi, cells were fixed and stained with phalloidin and DAPI. The acquired images were processed and analyzed using both a customized MatLab algorithm (see Materials and Methods, Fig. 2A,B; supplementary material Fig. S1B) and visual inspection. Using the fraction of cells with bars per well as a read-out, we identified the dsRNAs that caused a reduction in the number of cells with actin bar in the nucleus. Data was normalized per plate and the replicates were compared to each other (Fig. 2A,B). Hits (supplementary material Table S1) were then annotated using the Flybase (www.flybase.org) and DRSC (www.flyrnai.org) databases, revealing that the proteins were involved in a large spectrum of biological processes (Fig. 2C). Importantly, among the hits were proteins that we had previously shown to regulate the nuclear localization of actin, including Tsr, the Tsr phosphatase Slingshot (Ssh) and the small GTPase Ran (supplementary material Table S1) (Dopie et al., 2012), demonstrating that our assay was operational also in a genome-wide format. Of note, we also observed several potential hits that increased the number of nuclear actin bars (supplementary material Table S2). These hits are candidates for acting as negative regulators of nuclear actin export and depolymerization, complementing those identified earlier in the absence of exportin-6 co-depletion (Rohn et al., 2011). However, for the present work, we decided to concentrate on the hits displaying decreased numbers of nuclear actin bars.

We selected dsRNAs targeting 200 genes for retesting with the same dsRNA used in the genome-wide screen (supplementary material Table S3). At least one dsRNA from each biological function category (Fig. 2C) was included. Subsequently 113 putative hits (supplementary material Table S4) were retained for secondary screening using dsRNAs targeting different amplicons of the same genes. A total of 28 dsRNAs (supplementary material Table S5) also prevented the formation of the actin bar in the cell nucleus in this assay and the genes targeted were studied further.

### Identification of specific regulators of nuclear actin

We next aimed to exclude those hits that caused the disappearance of the nuclear actin bar in an unspecific manner (e.g. those not directly related to either actin nuclear import or polymerization). In some hits phalloidin staining seemed lower than in control cells. Reduced actin expression would explain also the lack of nuclear actin, and we therefore determined the levels of actin and Tsr proteins by western blotting. Indeed, some dsRNAs, such as those against Fs(1)h and CG40451, caused a notable decrease in actin protein levels, whereas others, such as CycE and Nej affected Tsr expression (supplementary material Fig. S2A–C). Our primary RNAi screening identified core components of the RNAi pathway, such as Argonaute 2 and Dicer 2, as putative hits in our assay (supplementary material Table S1). Their depletion can result in ineffective RNAi silencing and implies that dsRNAs that affect components of the RNAi machinery are potential false positives in our assay. However, none of the remaining hits significantly impaired the exportin 6 silencing efficiency (data not shown).

We have previously shown that depletion of Ran, which provides the energy gradient for active nucleo-cytoplasmic shuttling, can prevent the exportin 6 depletion induced nuclear accumulation of actin (Dopie et al., 2012). This suggests that some of the identified dsRNAs could affect nuclear–cytoplasmic shuttling in a general manner, and not be involved in actin-specific processes. To eliminate such dsRNAs from our candidates, we expressed an import substrate, NLS–GFP–pyruvate-kinase, which uses the SV40 large T-antigen nuclear localization signal (NLS) to enter the cell nucleus through importin α and β (Kalderon et al., 1984). We examined the nuclear–cytoplasmic distribution of this construct in cells treated with candidate dsRNAs compared to Ketel (the *Drosophila* importin-β) dsRNA-treated cells. Whereas depletion of Ketel efficiently retained the NLS–GFP–pyruvate-kinase in the cytoplasm, the majority of tested dsRNAs showed prominent nuclear localization of this construct (supplementary material Fig. S3A,B), indicating that they do not cause gross defects in general protein import into the nucleus. This assay demonstrated that CG6686, CycE, Mov34 and Probeta5 might have scored in our screen due to their unspecific effects on nuclear import (supplementary material Fig. S3B). Surprisingly, neither Nup358 nor Nup98, which are components of the nuclear pore complex (Hetzer and Wente, 2009) caused a significant block in nuclear import in this assay, suggesting that these nucleoporins might have a more specialized role in nuclear transport events, at least in the S2R+ cell model, or might influence nuclear actin by some other means. After excluding the unspecific hits, we were left with 19 proteins (Table 1), which were candidates for specifically regulating nuclear actin.

**Table 1.**
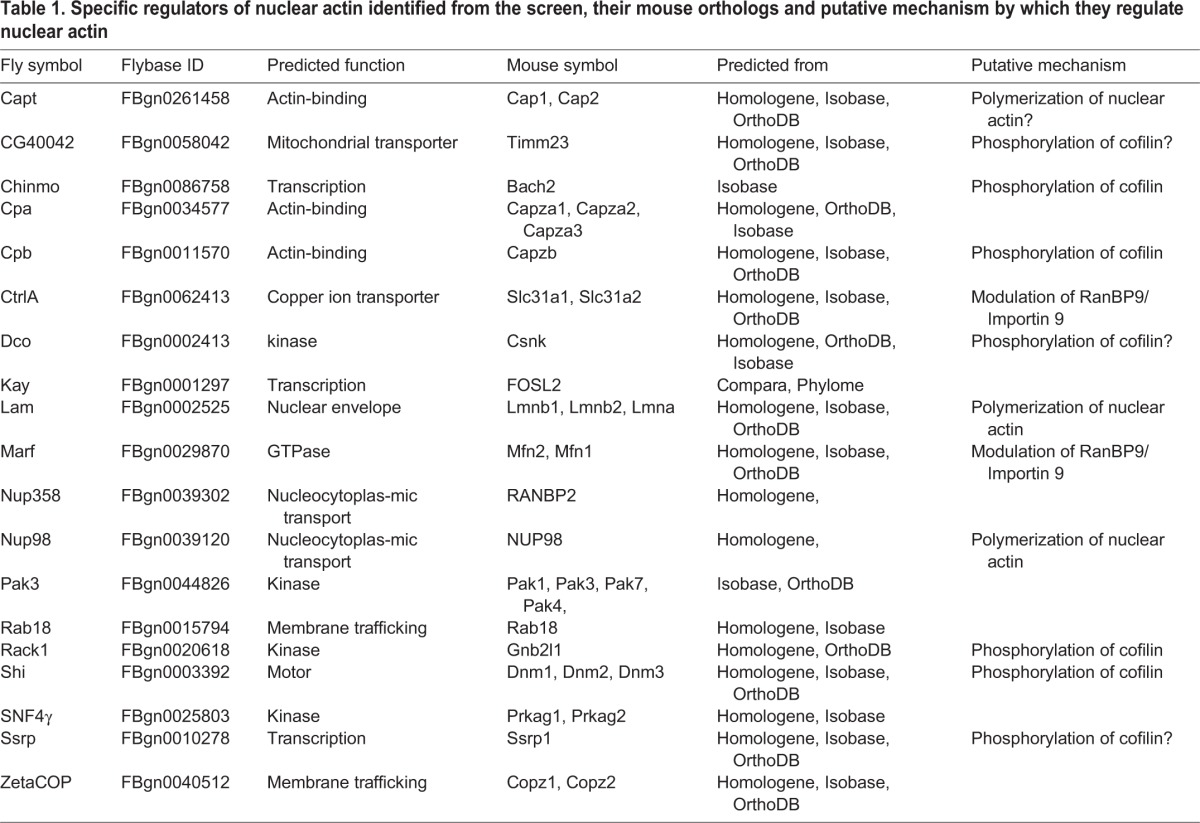
**Specific regulators of nuclear actin identified from the screen, their mouse orthologs and putative mechanism by which they regulate nuclear actin**

### Regulators of nuclear actin polymerization

**Fig. 3.**
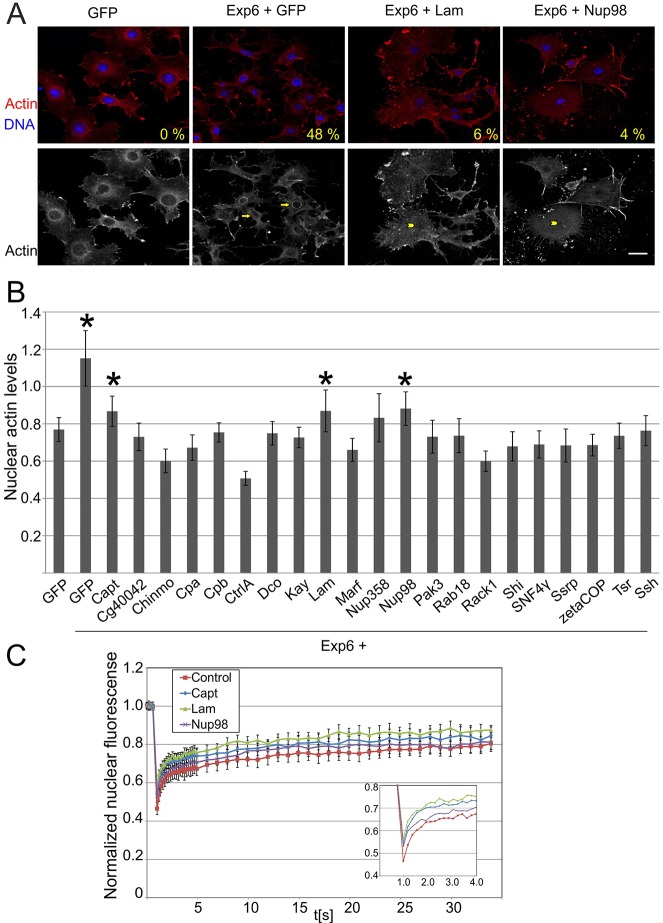
**Identification of putative regulators of nuclear actin polymerization.** (A) Representative images of S2R+ cells treated with indicated dsRNAs and stained with an actin antibody (red) and DAPI (blue) in a merged image (upper panel), or the actin alone (lower panel). Arrows indicate the nuclear actin bar, whereas the arrowheads indicate the nuclear accumulation of actin without an actin bar. The number in the upper panel indicates the percentage of cells with a nuclear actin bar as quantified from anti-actin antibody staining. Scale bar: 20 µm. (B) Quantification of nuclear actin signal. Data are mean±s.d. from 24–50 cells per condition. *P<0.05 increase in nuclear actin levels (Student's *t*-test). (C) Intranuclear mobility of GFP–actin measured by FRAP assay in S2R+ cells treated with indicated dsRNAs (Control, GFP dsRNA). The data are mean±s.d. of at least 11 cells per condition. The inset shows the first 4 s of the assay. Fitted parameters (see Materials and Methods) were: control *t*1=0.32±0.03, *t*2=12.2±1.0; Nup98 *t*1=0.27±0.05, *t*2=6.7±0.5; Lam *t*1=0.31±0.04, *t*2=9.6±0.9; Capt *t*1=0.32±0.03, *t*2=11.7±1.0.

The absence of the nuclear actin bar might be either due to insufficient nuclear accumulation of actin because of import defects or due to problems with forming the bar through actin polymerization. To distinguish between these possibilities, we stained cells co-depleted of exportin 6 and the hits with an actin antibody. The nuclear actin bar in exportin-6-depleted cells was also clearly seen with the antibody staining (Fig. 3A) and perfectly colocalized with the phalloidin-stained nuclear actin bar (supplementary material Fig. S3C). With the majority of the hits, the cell nucleus seemed rather empty of actin signal, but in a subset of hits, the actin antibody staining revealed a clear nuclear signal, but instead of a bar, this signal appeared rather smooth and did not form clear structures (Fig. 3A). Quantification of the nuclear actin signal revealed that cells depleted of Capt, Lam (the *Drosophila* lamin) and Nup98 had significantly increased levels of nuclear actin (Fig. 3B), despite the absence of the actin bar. These proteins are therefore candidates for regulating nuclear actin polymerization, whereas the other hits are more likely to operate in nuclear import of actin.

To further probe the requirement for Capt, Lam and Nup98 in nuclear actin polymerization, we measured the intranuclear mobility of GFP–actin by using a fluorescence recovery after photobleaching (FRAP) assay (Fig. 3C). The recovery curves exhibited two phases of recovery (see Materials and Methods for details). The first phase, which likely corresponds to actin monomer mobility, was essentially identical for all the conditions (0.27–0.32 s). However, cells depleted of Lam (9.6±0.9 s) or Nup98 (6.7±0.5 s) (mean±s.d.) both displayed significantly faster half times for the second phase of recovery, and thus higher mobility of GFP–actin than cells treated with control GFP dsRNA (12.2±1.0). In this assay, Capt-depleted cells (11.7±1.05) did not show significant differences compared to the control cells. Taken together, these results show that depletion of Lam and Nup98 increases actin mobility within the nucleus.

### New regulators of cofilin phosphorylation

We previously showed that the nuclear localization of actin is governed by the transport factor importin 9 and dephosphorylated Tsr (the *Drosophila* cofilin) (Dopie et al., 2012). Cells could therefore regulate the levels of nuclear actin by modulating the abundance and/or activity of these import mediators. We used quantitative RT-PCR (qRT-PCR) analysis to study RanBP9 (*Drosophila* importin 9) mRNA levels. Of the hits, depletion of only the copper ion transporter Ctr1A and mitochondrial protein Marf affected RanBP9 expression (supplementary material Fig. S4A). However, the mechanism by which these proteins operate here is not obvious.

**Fig. 4.**
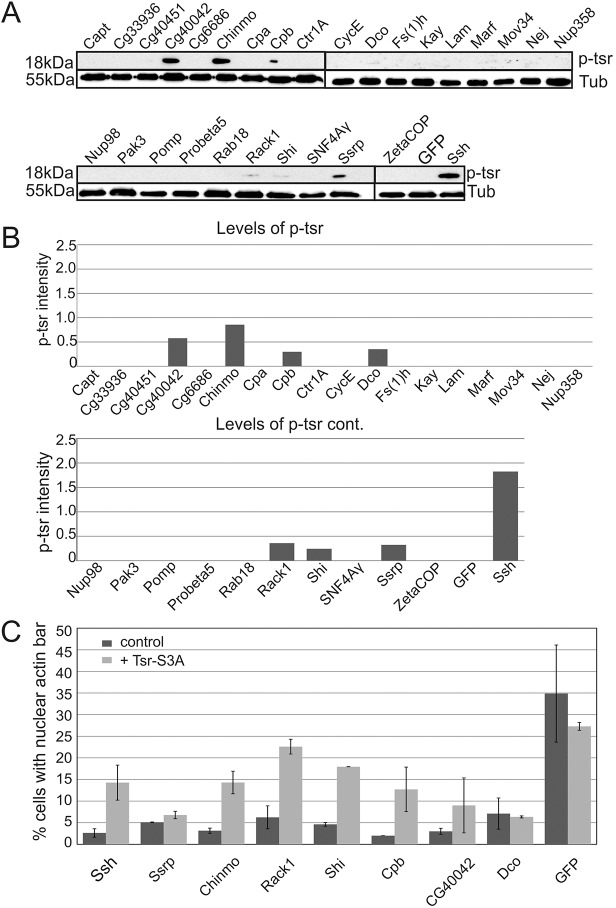
**A subset of hits affect cofilin activity.** (A) Western blot from S2R+ cells treated with dsRNAs as indicated, and probed for p-Tsr and tubulin. GFP and Slingshot (Ssh) dsRNAs were included as negative and positive controls, respectively. (B) Quantification of p-Tsr levels from the western blots. Data are the mean of two independent experiments. (C) Expression of Tsr-S3A restores the actin bar formation induced by exportin 6 depletion in a subset of hits associated with Tsr phosphorylation. Quantification of cells with nuclear actin bars after co-transfecting candidate dsRNAs with exportin 6 dsRNA and Tsr-S3A plasmid. Data are mean±s.d. from two independent experiments, with 15–117 cells quantified per sample.

Cofilins are actin filament-disassembling proteins (Bugyi and Carlier, 2010) and their actin-binding activity is regulated by phosphorylation on serine 3 (Agnew et al., 1995). Dephosphorylation of cofilins is essential for their role in the nuclear import of actin, and thus RNAi-mediated silencing of Ssh, the phosphatase of Tsr, results in the nuclear exclusion of actin (Dopie et al., 2012). Ssh also scored as a hit in our screen (supplementary material Table S1). To investigate whether some of our hits regulate cofilin activity, we analyzed the levels of phosphorylated Tsr (p-Tsr). Interestingly, depletion of CG40042, Chinmo, Cpb, Dco, Rack1, Shi and Ssrp led to a clear increase in the level of p-Tsr, whereas the total Tsr level remained unchanged (Fig. 4A,B; supplementary material Fig. S2A,B). Importantly, expression of an unphosphorylatable, and thus active Tsr mutant, Tsr-S3A, restored the bar formation upon Chinmo, Rack1, Shi and Cpb silencing (Fig. 4C), demonstrating that the increased p-Tsr associated with these hits is the likely underlying cause for the defect in actin nuclear import observed in our screen.

### Chinmo regulates Tesk expression

**Fig. 5.**
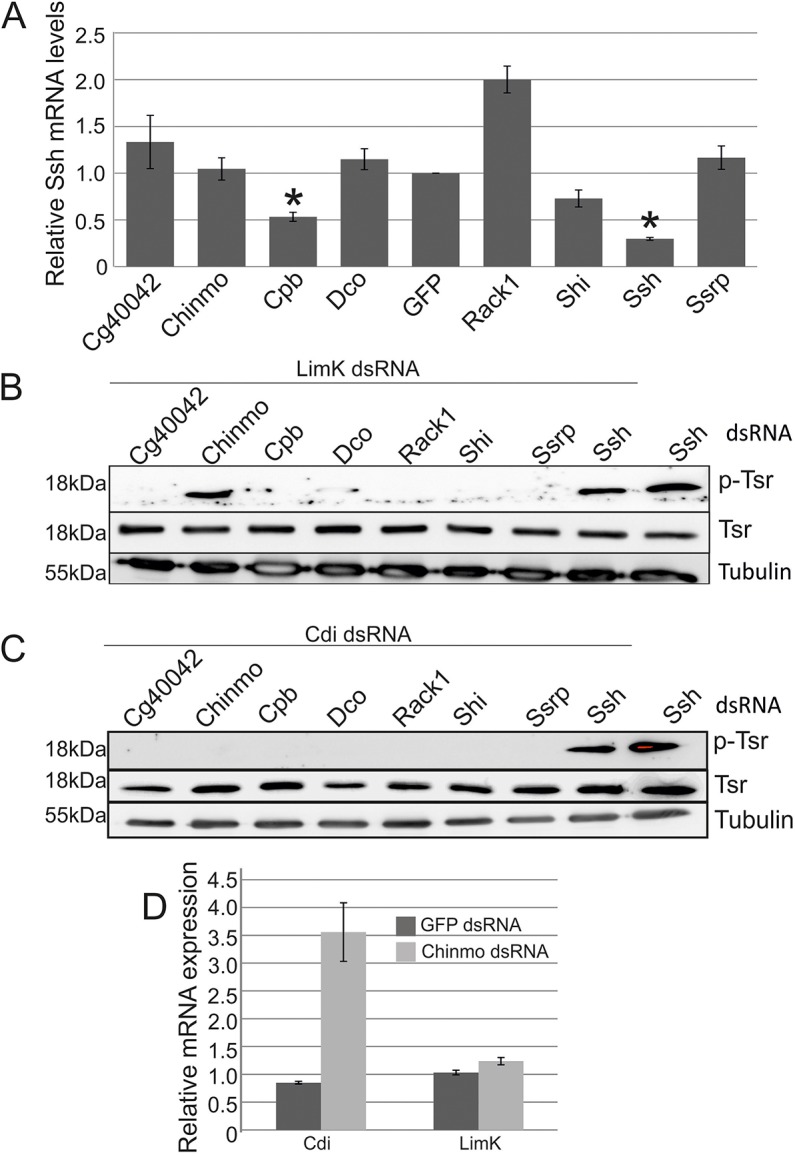
**Chinmo regulates cofilin phosphorylation through the expression of Cdi/Tesk.** (A) Quantification of the relative levels of Slingshot (Ssh) mRNA in cells treated with indicated dsRNAs. Data are mean±s.d. of two independent experiments. *P<0.05 (Student's *t*-test). (B,C) Representative western blots showing the levels of Twinstar (Tsr) and phosphorylated Tsr (p-Tsr) in cells treated with indicated dsRNAs. Tubulin was detected as a loading control. (D) Quantification of Tesk and Limk mRNA expression as measured by qRT-PCR in cells treated with either Chinmo or GFP dsRNA. Data are the mean±s.d. of at least two independent experiments.

To distinguish whether the hits that accumulated p-Tsr are involved in either phosphorylation or dephosphorylation of Tsr, we first measured the expression levels of Ssh, the major cofilin phosphatase (Niwa et al., 2002), by using qRT-PCR. Except for in Cpb-depleted cells, levels of Ssh did not decrease (Fig. 5A). Lim kinase (Limk) and Tes kinase (Tesk, known as Cdi in *Drosophila*) are the major cellular kinases responsible for Tsr/cofilin phosphorylation (Arber et al., 1998; Toshima et al., 2001). To elucidate if the identified proteins depend on the activity of these kinases to induce cofilin phosphorylation, we performed a co-depletion experiment and determined the levels of p-Tsr. Interestingly, depletion of either Limk or Cdi was sufficient to abolish the increased p-Tsr levels induced by CG40042, Cpb, Dco, Rack1, Shi and Ssrp depletion (Fig. 5B). However, p-Tsr remained in cells after Chinmo was co-silenced with Limk, and was lost only upon Cdi silencing (Fig. 5B,C). This indicates that Chinmo might specifically regulate cofilin phosphorylation through Tesk.

As Chinmo (Chronologically inappropriate morphogenesis) is a transcriptional repressor that contains a Bric-a-brac, Tramtrack, Broad Complex (BTB) domain (Zhu et al., 2006), we wondered whether it could regulate the expression of Cdi. Strikingly, the expression of Cdi was significantly upregulated in Chinmo-depleted cells as measured by qRT-PCR (Fig. 5D). This suggests that under normal conditions, Chinmo represses Cdi expression to maintain Tsr in an unphosphorylated, and thus active, state.

### Chinmo regulates nuclear actin levels *in vivo*

**Fig. 6.**
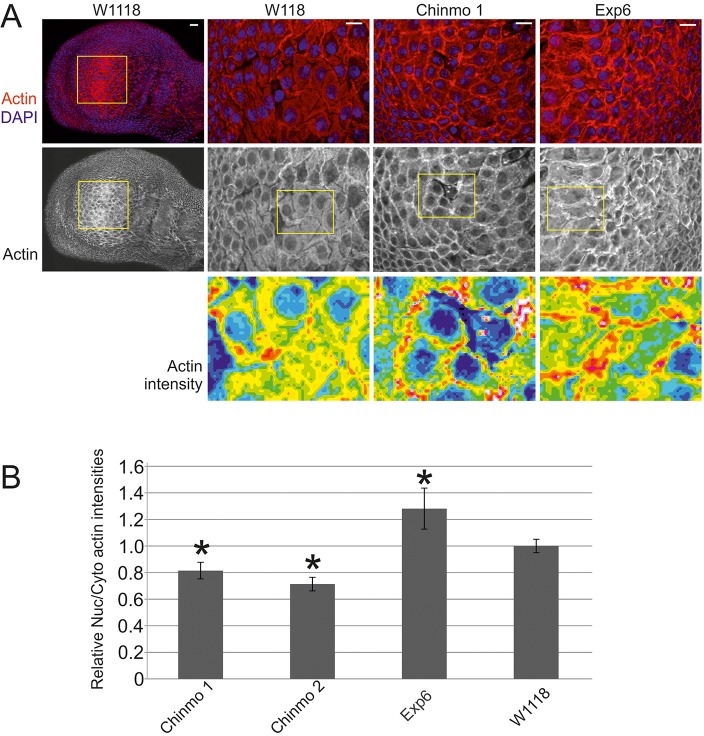
**Chinmo regulates nuclear actin levels also *in vivo*.** (A) Representative images of wing discs from the indicated fly strains stained with anti-actin antibody (top panel), and merged images (lower panel) showing actin (red) and DAPI (blue). The left panel shows the whole wing disc, and the region outlined with yellow shows the part of the imaginal wing pouch that was analyzed for the levels of nuclear actin (right panels). Scale bars: 10 µm. (B) Quantification of the ratio of the relative nuclear to cytoplasmic actin intensities in cells within the imaginal wing pouch normalized to the control sample. Data is mean±s.d. from at least 200 cells per sample from two biological replicates. Two independent UAS lines for Chinmo (Chinmo 1 and 2) were tested. *P<0.05 compared with control (W1118) (Student's *t*-test).

Although the nuclear actin bar represents an excellent model for robust scoring of nuclear actin phenotypes, it is, nevertheless, a non-native structure that is formed only upon depletion of specific factors. To confirm that our assay was capable of identifying factors that would regulate actin also *in vivo* in the context of whole organism, we investigated actin distribution within the pouch of the *Drosophila* wing imaginal disc, which we have found to be amenable for staining nuclear actin *in vivo*. We used the UAS-GAL4 system (Brand and Perrimon, 1993) to drive Chinmo or exportin 6 RNAi in the wing disc, and analyzed the nuclear and cytoplasmic actin distribution (Fig. 6A,B). Strikingly, both RNAi strains for Chinmo displayed decreased nuclear actin levels as compared to wild-type flies. As expected, exportin 6 silencing resulted in increased nuclear actin (Fig. 6A,B), but did not lead to formation of a similar nuclear actin bar as observed in cultured S2R+ cells (Fig. 1A). This could be because the silencing efficiency was lower *in vivo*, or, alternatively, the cultured cells could be lacking some regulatory mechanisms that prevent ectopic actin polymerization in tissues. Our results therefore uncover Chinmo as a factor capable of regulating nuclear actin levels in model organisms, and highlight that the appropriate regulation of cofilin activity is important in maintaining nuclear actin *in vivo*.

### Nuclear actin import regulators are evolutionary conserved

**Fig. 7.**
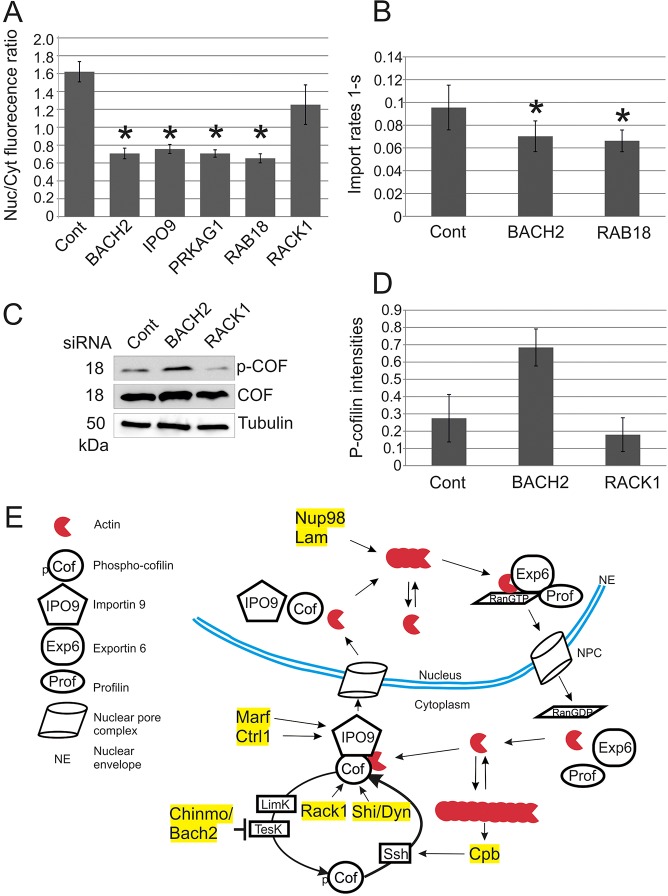
**Nuclear actin regulating proteins are evolutionarily conserved.** (A) Quantification of the ratio between nuclear and cytoplasmic fluorescent intensities of actin measured with ImageJ. See supplementary material Fig. S4B for representative images. Data are the mean±s.d. from three independent experiments with at least 30 cells quantified per sample. *P<0.05 (Student's *t*-test). (B) Nuclear import rate of GFP–actin as measured with a FRAP assay in cells treated with indicated siRNAs. Data are the mean±s.d. from 17–18 cells per condition. *P<0.05 (Student's *t*-test). (C) Representative western blot showing phosphorylated cofilin (p-COF) and cofilin (COF) from NIH3T3 cell lysates treated with indicated siRNAs. Tubulin was used as a loading control. (D) Quantification of phosphorylated cofilin (p-cofilin) levels from western blots. Data is the mean±s.e.m. from three experiments. (E) A working model of the nucleo-cytoplasmic transport of actin with the new nuclear import regulators. Nuclear export of actin is mediated by exportin 6 (Exp6), profilin (Prof), and Ran in its GTP-bound form (RanGTP). In the cytoplasm, actin monomers exist in dynamic equilibrium with actin filaments, and cofilin (Cof) severs and depolymerizes actin filaments to maintain the actin monomer pool. The actin-binding activity of cofilin is regulated by phosphorylation mediated by the Lim (LimK) and Tes (TesK) kinase, whereas dephosphorylation is mediated by Slingshot (Ssh). Unphosphorylated cofilin and importin 9 (IPO9) bind actin monomers in the cytosol and the complex translocates to the nucleus. Copper transporter 1A (Ctr1A) and mitochondrial assembly regulatory factor (Marf) maintain expression levels of importin 9. Rack1 and dynamin (Shi/Dyn) maintain cofilin in the active unphosphorylated state through an uncharacterized mechanism. Chinmo/Bach2 represses the expression of TesK to promote accumulation of unphosphorylated cofilin, whereas Cpb promotes the expression of Ssh, in a process that might be sensitive to the polymerization status of actin. Nup98 and Lam are candidates for regulating polymerization of nuclear actin.

To address the evolutionary implications of our findings, we examined whether silencing the mammalian orthologues of the identified *Drosophila* genes would affect nuclear import of actin. We selected a subset of hits including Chinmo (Bach2), Rab18, Rack1 (Gnb2l1) and SNF4Aγ (Prkag1) and analyzed the specific siRNAs targeting these proteins for their ability to prevent the nuclear accumulation of endogenous actin after exportin 6 silencing in mouse NIH 3T3 cell line. Silencing of Bach2, Prkag1 and Rab18 efficiently suppressed the nuclear accumulation of actin induced by exportin-6 depletion (Fig. 7A; supplementary material Fig. S4B). However, we failed to reproduce the phenotype of Rack1 in mammalian cells. Moreover, depletion of either Bach2 or Rab18 significantly decreased the nuclear import rate of actin (Fig. 7B), as measured with a fluorescence recovery after photobleaching assay (Dopie et al., 2012; Skarp and Vartiainen, 2013), demonstrating that these proteins are indeed involved in nuclear entry of actin. Finally, silencing of Bach2 in NIH 3T3 cells resulted in increased levels of phosphorylated cofilin (Fig. 7C,D), similar to what is observed upon Chinmo depletion in S2R+ cells (Fig. 4). These results suggest that, like nuclear export regulators (Rohn et al., 2011), nuclear import regulators of actin are also evolutionary conserved, underscoring the importance of nuclear actin level control in different organisms.

## DISCUSSION

Actin has been linked to processes spanning the whole gene expression cascade (Grosse and Vartiainen, 2013), and active nuclear import of actin is required to sustain maximal transcriptional activity of the cell (Dopie et al., 2012). Given that altered nuclear actin levels have been linked to cell quiescence (Spencer et al., 2011) and differentiation (Xu et al., 2010), which are very fundamental biological processes, it is fair to assume that the nucleo-cytoplasmic shuttling of actin is very tightly regulated, and perhaps the target of several cellular signaling pathways. The fact that actin uses an active transport mechanism for both nuclear import (Dopie et al., 2012) and export (Stuven et al., 2003), despite being at the limit for passive diffusion through the nuclear pore, already supports this hypothesis. Polymerization of actin into helical filaments is fundamental for its functions in the cytoplasm, and one of the most tightly regulated processes in the cell. Understanding the mechanisms that regulate nuclear actin polymerization is one key to deciphering the exact nature of nuclear actin polymers. Our genome-wide RNAi screen uncovered 19 new regulators of nuclear actin polymerization and import (Table 1), thus expanding the cellular processes impinging on nuclear actin (Fig. 7E).

Our assay was based on ‘rescuing’ the formation of a phalloidin-stainable nuclear actin bar caused by exportin 6 depletion. The identified proteins are then candidates for regulating either nuclear import of actin or the actual formation of the nuclear actin bar. Our actin antibody staining demonstrates that we identified both types of proteins in our screen. Depletion of Capt, Lam, or Nup98 together with exportin 6 resulted in clear nuclear accumulation of actin in the absence of the actin bar, indicating that these proteins are candidates for regulating nuclear actin polymerization. Moreover, depletion of Lam and Nup98 also increased nuclear mobility of actin in a FRAP assay (Fig. 3C). Of interest, Lam is the *Drosophila* lamin, a component of the nuclear lamina underlying the nuclear envelope. Loss of or mutations in *LMNA* in mammalian cells result in impaired nuclear actin polymerization due to mislocalization of emerin, an ABP of the inner nuclear membrane (Ho et al., 2013). Moreover, a recent study has demonstrated that there is transient actin polymerization, which is dependent on the linker of cytoskeleton and nucleoskeleton complex (LINC) and emerin, upon cell spreading (Plessner et al., 2015). Whether Lam operates through similar mechanisms in *Drosophila* cells remains to be determined; however, emerin is perhaps not involved here because it did not score as a hit in our screen. Nevertheless, our study reinforces the notion that the nuclear lamina might play a pivotal role in regulating nuclear actin dynamics. In addition to nucleo-cytoplasmic transport, Nup98 has been implicated in gene expression, and chromosomal rearrangements affecting its gene are associated with a wide range of malignancies (Gough et al., 2011). In our experimental system, neither depletion of Nup98 nor the other nucleoporin hit from our screen, Nup358, caused a general block in nuclear import (supplementary material Fig. S3B). It is therefore possible that these factors are involved in nuclear trafficking of some specific actin regulators. Alternatively, Nup98 could regulate the expression of some key nuclear actin polymerization factors. Somewhat surprisingly, our assay did not reveal any canonical actin polymerization promoters, such as Arp2/3 complex or formins, that have been linked to nuclear actin and gene expression processes (Yoo et al., 2007; Baarlink et al., 2013). This could be due to the fact that these proteins also have important roles in the cytoplasm that will dominate their RNAi phenotypes. Alternatively, there might be functional redundancy among actin nucleators in the nucleus. Finally, one limitation of our assay is that once the exportin 6 bar is fully formed, we have noticed that it is rather stable. Hence proteins that are stable, and thus depleted by RNAi slower than exportin 6, will likely be missed with our assay.

Our present study strengthens the notion that appropriate regulation of nuclear actin levels through controlled nucleo-cytoplasmic shuttling is an evolutionary conserved phenomenon, because both nuclear import regulators (this study) and export regulators (Rohn et al., 2011) have been conserved from fly to mammals. Receptor for activated C kinase 1 (Rack1, also known as Gnb2l1 in mammals) was the only protein of the four that we tested that failed to prevent the nuclear accumulation of actin induced by exportin 6 depletion in mouse cells. Rack1 is involved in many signaling complexes (Mamidipudi et al., 2004; Doan and Huttenlocher, 2007; Jia et al., 2013), and it is therefore possible that in mammals there is more redundancy in the signaling pathways utilizing this protein than in the fly.

Nuclear actin levels can be regulated at several different steps (Dopie et al., 2012; Huet et al., 2013), and thus the import hits from our screen could act at any of these points. Of the identified hits, only Ctr1A and Marf affected the levels of RanBP9/importin 9 (supplementary material Fig. S4A). Ctr1A is the primary copper transporter in *Drosophila*, responsible for copper uptake across the plasma membrane and required for fly development (Turski and Thiele, 2007). Marf, by contrast, is an essential transmembrane GTPase that mediates mitochondrial fusion (Dorn et al., 2011). The mechanism by which these proteins could regulate RanBP9/importin 9 expression is therefore not very obvious. How the activity of importin 9 might be regulated is not known, and therefore some of the hits could operate this way.

Several identified hits affected the cofilin protein Tsr, either by reducing its protein levels (supplementary material Fig. S2A,B) or by modulating its activity through phosphorylation (Fig. 4). Cofilin proteins have a dual role in nuclear import of actin: as the main actin filament disassembly and severing protein (Lappalainen and Drubin, 1997), it maintains the transport competent monomer pool, and at the same time, it mediates the interaction between importin 9 and actin (Dopie et al., 2012). Of our cofilin regulators, to our knowledge only Rack1 has been linked to cofilin phosphorylation previously (Cao et al., 2011). Although expression of the constitutively active Tsr-S3A mutant restored the actin bar formation induced by exportin 6 depletion in Chinmo-, Rack1-, Shi- and Cpb-depleted cells, it failed to do so in Ssrp-, CG40042- and Dco-depleted cells. This suggests that although Ssrp, CG40042 and Dco depletion cause increased p-Tsr levels, it is likely not the reason why they scored as hits in our assay. Importantly, none of the hits that caused increased phosphorylation of cofilin displayed increased levels of nuclear actin (Fig. 3). This demonstrates that in the system used here, cofilin operates mainly at the level of nuclear import of actin, and not at the level of nuclear actin bar formation. This supports our previous results that cofilin is required also for the nuclear accumulation of an unpolymerizable actin mutant, actin-R62D (Dopie et al., 2012).

We found examples of both negative regulators of cofilin kinases and positive regulators of cofilin phosphatase among our hits. Capping protein beta (Cpb) is a subunit of the heterodimeric protein that caps the barbed ends of growing actin filaments and prevents filament elongation (Amatruda et al., 1990). The Cpb-depleted cells displayed decreased levels of the cofilin phosphatase Ssh (Fig. 5A), which likely explains the accumulation of phosphorylated cofilin in these cells. Actin filaments seem to regulate the activity of Ssh (Soosairajah et al., 2005). Given that capping protein regulates the growth of actin filaments, it is possible that there is an indirect feedback mechanism through the actin cytoskeleton to Ssh expression. Of note, the other subunit of capping protein, Cpa, was also identified as a hit in our screen (Table 1), but failed to elicit accumulation of p-Tsr (Fig. 4). Interestingly, with the exception of Chinmo, we could prevent the accumulation of p-Tsr by co-depleting either Limk or Tesk (Fig. 5B,C). This indicates that in S2R+ cells, the activities of these two kinases are interdependent. Many upstream signaling pathways are known to regulate Limk, but the signaling pathways leading to Tesk activation are less characterized (Mizuno, 2013). It is possible that one of our newly characterized Tsr/cofilin regulators could act to bridge the signaling to these two kinases.

Chinmo, which modulates cofilin activity by regulating Cdi/Tesk expression (Fig 4; Fig.5C,D), is a transcriptional repressor that governs neuronal development in *Drosophila* (Zhu et al., 2006) and has been suggested to be an important effector of JAK–STAT pathway in various developmental and pathological conditions (Flaherty et al., 2010). As our data demonstrate that depletion of Chinmo also leads to a decrease in the levels of nuclear actin *in vivo* (Fig. 6A,B), it will be interesting to study in the future if nuclear actin, which has also been linked to cell fate decisions, also functions as a downstream effector of JAK–STAT–Chinmo axis in the context of a whole organism. Decreased nuclear actin in Chinmo-depleted wing discs also underscores the importance of appropriate regulation of cofilin activity to maintain nuclear actin levels *in vivo*.

It is rather intriguing that out of the 19 ‘specific’ hits, three (zetaCOP/Copz1, Rab18 and Shi/dynamin) are all connected to membrane trafficking. How this relates to nuclear transport of actin remains to be investigated, but especially the role of the small GTPase Rab18, which has been linked to ER–Golgi trafficking (Dejgaard et al., 2008), is intriguing, given that its function seems to be also conserved in mammalian cells (Fig. 7). Another protein with a conserved role in nuclear localization of actin is the regulatory subunit of the AMP-activated protein kinase (AMPK), SNF4Aγ/Prkag1 (Fig. 7), which is part of the central AMPK enzyme that monitors cellular energy status. AMPK is activated in response to decreased ATP levels, promoting energy-producing pathways and inhibiting energy-consuming processes (Hardie, 2007). AMPK has also been linked to actin cytoskeleton remodeling (Miranda et al., 2010). The mechanism by which AMPK impinges on nuclear import of actin needs to be clarified, but it is intriguing that nuclear accumulation of actin has been reported upon ATP depletion (Pendleton et al., 2003) as well as upon many other types of cellular stresses (Nishida et al., 1987; Iida et al., 1992). It is tempting to speculate that activation of AMPK in these conditions ‘pushes’ actin to the nucleus to somehow modulate gene expression.

Taken together, our genome-wide RNAi screen has identified 19 new regulators of nuclear actin. Four of these factors, Chinmo/Bach2, Rack1, Shi/dynamin and Cpb operate by regulating the phosphorylation status of Tsr/cofilin, whereas Ctr1A and Marf, through an unknown mechanism, control RanBP9/importin 9 levels. Lam and Nup98, by contrast, are new candidates for regulating nuclear actin polymerization (Fig. 7E). How the remaining hits impinge on nuclear actin remains to be investigated. However, the hits have been linked to a wide variety of processes ranging from membrane trafficking (e.g. Rab18) to cellular energy metabolism (SNF4Aγ/Prkga1). This suggests that regulation of nuclear actin is linked to many different cell biological processes.

## MATERIALS AND METHODS

### Primary genome-wide RNAi screening

The Drosophila RNAi Screening Center (Harvard Medical School) library 2.0 (http://www.flyrnai.org/DRSC-DRS.html) was used for the primary screen. This library contained about 13,900 genes that cover over 95% of the entire *Drosophila* genome and the screen was performed in duplicate in 384-well black thin-bottomed screening plates (PerkinElmer). In total, 128 pre-aliquoted screening plates were used. Before cells were seeded in plates, water in the designated ‘empty wells’ was replaced with 25 ng of dsRNA against Tsr. S2R+ cell suspension, in serum-free medium, was mixed with exportin 6 dsRNA to a final concentration of 0.025 µg/µl. About 10 µl of dsRNA-containing cell suspension was quickly seeded (8000 cells/well) in the screening plates and incubated at 25°C. After 30 min, 35 µl of complete Schneider's medium was added and cells were incubated in a humidified chamber at 25°C. After 5 days of dsRNA treatment, 50 µl of 8% paraformaldehyde (PFA) was added to wells and fixed for 20 min. Cells were washed with PBS and permeabilized using 0.2% Triton X-100 (Sigma). Cells were stained with Alexa-Fluor-488-labeled phalloidin (Molecular Probes) and DAPI (Sigma) for 30 min and washed three times with PBS. Approximately 50 µl of PBS was left on each well to prevent cells from drying out during imaging. Four fields were imaged per well using the 20×/ 0.7 NA water immersion objective of the Perkin Elmer Evotec Opera confocal microscope (Perkin Elmer). A script in the Acapella script collection was used to export FLEX file images into TIF files for image processing and analysis.

### Image processing, data analysis and hit identification of the RNAi screen

Images were processed and analyzed using a customized automated MatLab algorithm, and data was scored as the ratio of cells with nuclear actin bars per well (Fig. 2A,B; supplementary material Fig. S1B). For the data analysis, individual plates were median centered by dividing the well data by the plate median to correct for drifts. Then the median and the median absolute deviation (MAD) was determined for the entire dataset and absolute deviations from the median were calculated for individual wells. ‘Wells of interest’ were defined as dsRNAs with replicate well values≤median−2MAD (2MAD) (Zhang et al., 2006). We chose 2MAD because this covered over 95% of all positive controls in the screen (Fig. 2B). About 1000 wells of interest were selected for further consideration, and the targeted protein provided by DRSC (supplementary material Table S1). Images were visually inspected and dsRNAs that affected cell viability were eliminated.

### Retesting and secondary validations

Hits were manually annotated based on function, using the publicly available Flybase (http://flybase.org/) and DRSC databases (www.flyrnai.org). About 200 dsRNAs were retested (supplementary material Table S2), out of which 113 also had an effect in this second screen and were retained for secondary screening (supplementary material Table S3). dsRNA against different amplicons of the hits were provided by the DRSC and their effect on the nuclear localization of actin was tested in 96-well black clear-bottomed plates (Perkin Elmer). Images for the secondary screen were acquired using the 20×0.45NA objective of Thermo Scientific CellInsight (Thermo Scientific) and were analyzed with the automated algorithm as described above and visual inspection.

### Antibodies and DNA constructs

The antibodies used in this study include: rabbit polyclonal anti-actin (A2103; Sigma), mouse anti-α-tubulin (Sigma), rabbit anti-p-Tsr (Signalway Antibody) and rabbit anti-Tsr (kind gift from James Bamburg, Department of Biochemistry & Molecular Biology, Colorado State University, USA) as well as secondary horseradish peroxidase (HRP)-conjugated anti-mouse-IgG and anti-rabbit-IgG antibodies (Sigma); Alexa-Fluor-conjugated anti-mouse-IgG and anti-rabbit-IgG antibodies (Molecular probes). pAW-NLS-GFP-PK was generated by PCR from a pEGFP-C1 plasmid containing NLS–GFP–pyruvate-kinase and cloned into the pAW gateway destination vector (Invitrogen). pAGW-actin (encoding GFP–actin) was created by cloning *Drosophila* actin-5C sequence to a Gateway-entry vector, and then to pAGW. The Twinstar S3A mutant (Tsr-S3A), was generated using primers (forward, 5′-ATGGCT**GCT**GGTGTAACTGTGTCTGATGTC-3′ and reverse, 5′-GACATCAGACACAGTTACACC**AGC**AGCCAT-3′) carrying the serine 3 to alanine mutation (bold nucleotides) in an *in vitro* mutagenesis reaction using a wild-type Tsr construct cloned into the pAWH destination vector (Invitrogen) as a template.

### Cell culture and RNAi

*Drosophila* S2R+ cells were maintained in a humidified chamber at 25°C, in Schneider's medium supplemented with 10% heat-inactivated fetal bovine serum (FBS) and penicillin-streptomycin (GIBCO). NIH 3T3 cells were cultured in Dulbecco's modified Eagle's medium (DMEM; Sigma) containing 10% FBS and penicillin-streptomycin (GIBCO) and maintained at 37°C and 5% CO_2_ until needed for experiments. dsRNAs were synthesized and purified using the MEGAscript^®^ T7 Kit and NucAway™ Spin columns (Applied Biosystems), respectively. Exportin 6 and importin 9 siRNAs have been described previously (Dopie et al., 2012). The other siRNAs were from Sigma, and the targeted sequences were: BACH2, 5′-GCAUUGACCCAGUACCCUA-3′; PRKAG1, 5′-CCUAGAUGUGUCUGUGACA-3′; RAB18, 5′-GCAAGCAUUCUAUGUUGUU-3′; and RACK1, 5′-GUUAUGGAAUACUCUGGGU-3′.

RNAi experiments and immunostainings were carried out as described previously (Dopie et al., 2012). Cells were imaged using 63×1.4 NA objective of Axio Imager M2 equipped with AxioCam HRm camera and AxioVision software (Zeiss) or TCS SP5 MP SMD FLIM equipped with a 63× (NA 1.3) oil objective and LAS AF software (Leica) or Zeiss LSM 700 equipped with LCI Plan-Neofluar 63× (NA 1.3) water and glycerol objective and ZEN software. Where necessary, nuclear and cytoplasmic fluorescent intensities were measured using ImageJ (NIH).

Nuclear import rates of GFP–actin were measured as described previously (Dopie et al., 2012; Skarp and Vartiainen, 2013). FRAP assays to measure intranuclear mobility of GFP–actin were performed on a Leica TCS SP5II HCS A confocal microscope using FRAPbooster. Laser power (Ar 488 nm; 35 mW) was set to 80%, and 0.5% was used for imaging. The images were acquired using 12-bit, 128×128 format, 1400 Hz, line averaging 2 and zoom 15.5, resulting in 0.193 s per frame. After four pre-bleach images, a circle with the diameter of 1.5 µm was bleached with 100% laser power with the zoom in option. The recovery was followed as fast as possible for the first 5 s, and then at 1 s intervals. For data analysis, the average of pre-bleach values was set to 1. Data was fitted to *y*=*y*0+A1×(1−exp(−*x*/*t*1))+A2×(1−exp(−*x*/*t*2)). Data is a mean from experiments performed on 11–18 cells from two independent experiments.

Flat-embedding for electron microscopy was performed as previously described (Seemann et al., 2000; Jokitalo et al., 2001) with the exception of fixing the cells with 2% glutaraldehyde, 2% paraformaldehyde in 0.1 M sodium cacodylate buffer, pH 7.4, and performing staining with 1% uranyl acetate, 0.3 M sucrose in distilled water en-block at +4°C for 1 h prior to dehydration. Sections (90-nm thick) were cut parallel to the coverslip, post-stained with uranyl acetate and lead citrate, and imaged with a Jeol JEM-1400 (Jeol Ltd., Tokyo, Japan) microscope, equipped with a Gatan Orius SC 1000B bottom mounted CCD-camera (Gatan Inc., USA), operating at 80 kV.

For western blotting, proteins were separated in 12% SDS-PAGE gels and transferred onto nitrocellulose membranes. The following primary antibodies and respective dilutions were used: mouse anti-α-tubulin (Sigma, dilution 1:10,000), rabbit anti-p-Tsr/cofilin (Signalway Antibody, dilution 1:1000), rabbit anti-Tsr (kind gift from James Bamburg, dilution 1:15,000), rabbit anti-cofilin (dilution, 1:1000). Secondary antibodies include: anti-mouse-IgG and anti-rabbit-IgG HRP-conjugated antibodies (Sigma, dilution 1:10,000). Immunoblots were developed using the LAS 3000 (Fujifilm) and where necessary, bands were quantified using ImageJ. For analysis of Tsr and actin levels (supplementary material Fig. S2), the experiment was performed at least four times, and due to the variability in the assay, the two most complete sets of blots were quantified. The trend was, nevertheless, the same in all experiments.

### Real-time quantitative PCR

RNAi was performed as described above and after 5 days, total mRNA was extracted using the Nucleospin RNA II kit according to the manufacturer's protocol (Macherey-Nagel). 500 ng of total mRNA was used for reverse transcription PCR using the Thermo Scientific RT-PCR kit and random primers (Thermo Scientific). Quantitative PCR was carried out using the Bio-Rad CFX machine (Bio-Rad) and SYBR green qPCR reagent (Thermo Scientific). The following primers were used: Gapdh_forward, 5′-AAGGGTGCGTCCTATGATGA-3′; Gapdh_reverse, 5′-GCCAAACTCGTTGTCGTACC-3′; Tesk_forward, 5′-GTCACACATCGTACAACCGG-3′; Tesk_reverse, 5′-GTCTTATCTTCTGCGTGGCG-3′; Limk_forward, 5′-GGCCGTAGTAATCCTCACGA-3′; Limk_reverse, 5′-TTGTCGGGGTCAACTACTGC-3′; Ssh_forward, 5′-CGTCAGCCACTACGCTGTAA-3′; Ssh_reverse, 5′-TTTGGCTTCGAAATTTTGCT-3′; RP49 forward, 5′-AGGGTATCGACAACAGAGTG-3′; and  RP49 reverse, 5′-CACCAGGAACTTCTTGAATC-3′.

Relative expression levels were calculated by the comparative C_T_ method, normalizing to Gapdh or RP49 cDNA with the equation 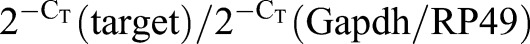.

### Fly strains, immunostaining and analysis of *Drosophila* imaginal discs

We used the GAL4-UAS system for RNAi and the fly lines include UAS-Dcr2-Nubbin-GAL4 (a gift from Dr Osamu Shimmi, Institute of Biotechnology, University of Helsinki, Finland) and the following UAS lines: FBst0026777 (Chinmo-1) and FBst0033638 (Chinmo-2) for Chinmo, FBst0032347 for exportin 6. These are also described in flybase (http://flybase.org). W1118 was a kind gift from Minna Poukkula (Institute of Biotechnology, University of Helsinki, Finland). Wing discs were dissected in PBS and fixed with 4% paraformaldehyde for 30 min at room temperature. Tissues were permeabilized in 0.5% Triton X-100 in PBS (PBT) for 10 min and blocked with 5% bovine serum albumin (BSA) in PBT for 1 h. Tissues were incubated with rabbit anti-actin primary antibody (Sigma) (A2103, dilution 1:250) overnight at 4°C. Tissues were washed in PBT for 1 h, and then incubated with Alexa-Fluor-labeled rabbit secondary antibodies and DAPI for 1 h at room temperature. Tissues were washed with PBT for 1 h and mounted in glycerol containing n-propylgallate (2.5%). Images were acquired using the Leica TCS SP5 confocal microscope, with a 63×1.3 NA objective and LAS AF software. Nuclear and cytoplasmic fluorescence intensities were measured using CellProfiler.

### Statistics

All error bars are based on standard deviation and the statistical analyses were performed in Excel using a two-tailed Student's *t*-test. Significance was taken as *P*<0.05.

## Supplementary Material

Supplementary Material
