# Supplementary material for: Genome-wide RNAi screen for nuclear actin reveals a network of cofilin regulators
Source: J Cell Sci. 2015 Jul 1;128(13):2388–400. doi: 10.1242/jcs.169441 (PMC4510847; doi:10.1242/jcs.169441)
Supplement: Supplementary Material [file supp_128_13_2388__index.html]

Genome-wide RNAi screen for nuclear actin reveals a network of cofilin regulators — Supplementary Material 

# Genome-wide RNAi screen for nuclear actin reveals a network of cofilin regulators

## JCS169441 Supplementary Material

**Files in this Data Supplement:**

- **Supplementary Material**
