# Supplementary material for: Genome-wide RNAi screen for nuclear actin reveals a network of cofilin regulators
Source: J Cell Sci. 2015 Jul 1;128(13):2388–400. doi: 10.1242/jcs.169441 (PMC4510847; doi:10.1242/jcs.169441)
Supplement: Supplementary Material [file supp_128.13.2388_JCS169441.pdf]

Supplementary Figure S1

A

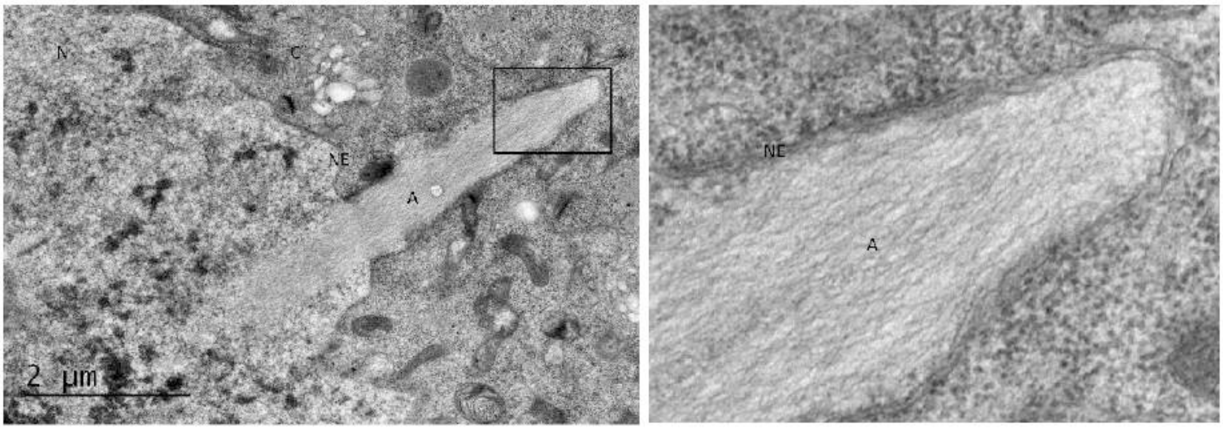

B

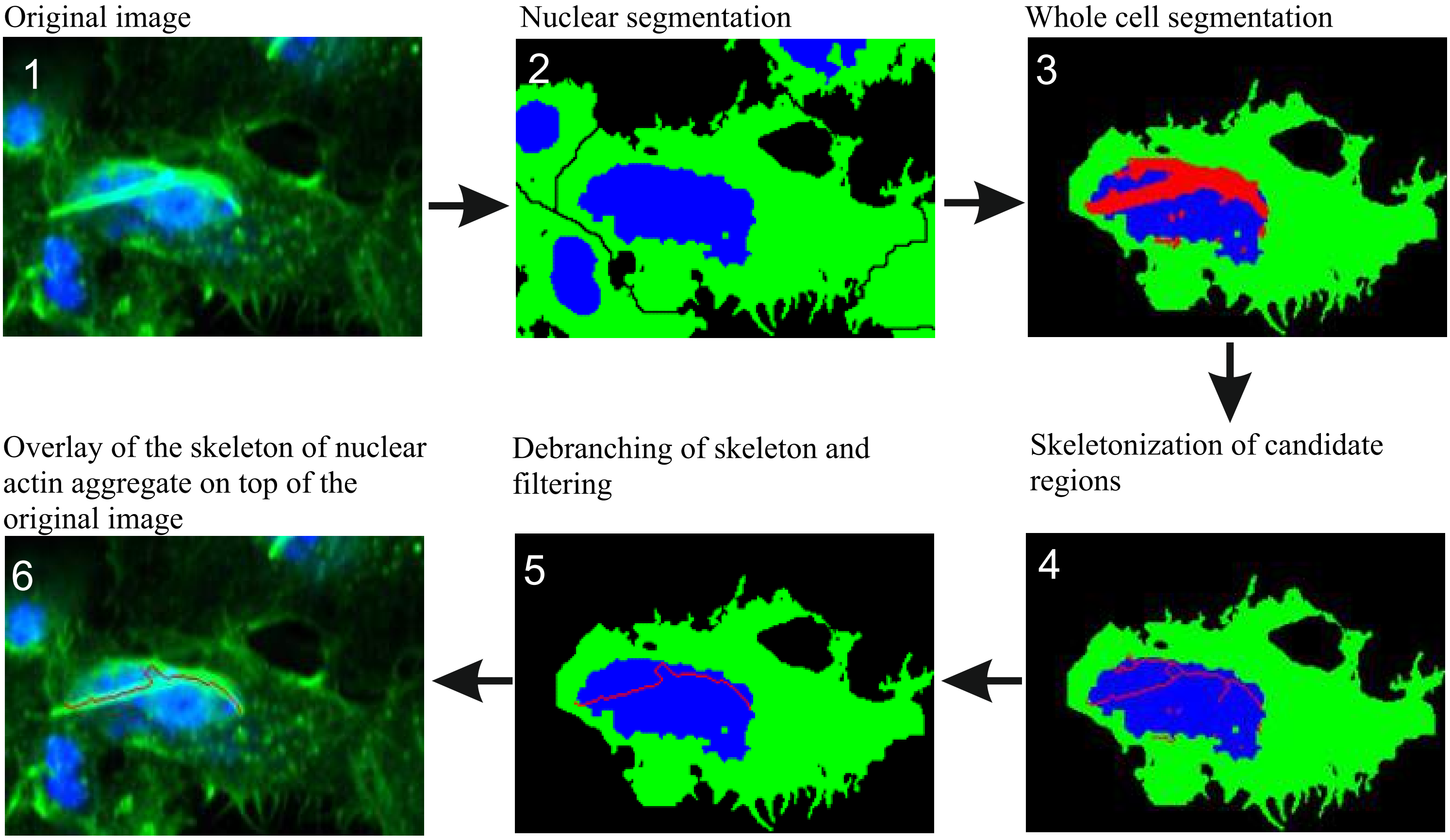

(A) Electron microscopy image showing exportin 6 dsRNA treated Drosophila S2R+ cell. Actin bar (A) protruding from the nucleus (N) and pushing the nuclear envelope (NE) to the cytoplasm (C) are indicated. Boxed area is shown with higher magnification on the right.

(B) A workflow diagram of the image analysis process for the identification of rod-shaped nuclear actin bars. (1) Original image with DAPI label for nuclei in blue and actin label in green. (2) Overlay of nuclear segmentation mask in blue and whole cell segmentation mask in green. (3) Overlay of nuclear actin bar mask in red on top of nuclear and whole cell masks. (4) Skeletonization of nuclear actin bar mask. (5) Cleaned up skeleton of nuclear actin bar after removing all the side branches. (6) Overlay of the skeleton of nuclear actin bar on top of the original image.

Supplementary Figure S2

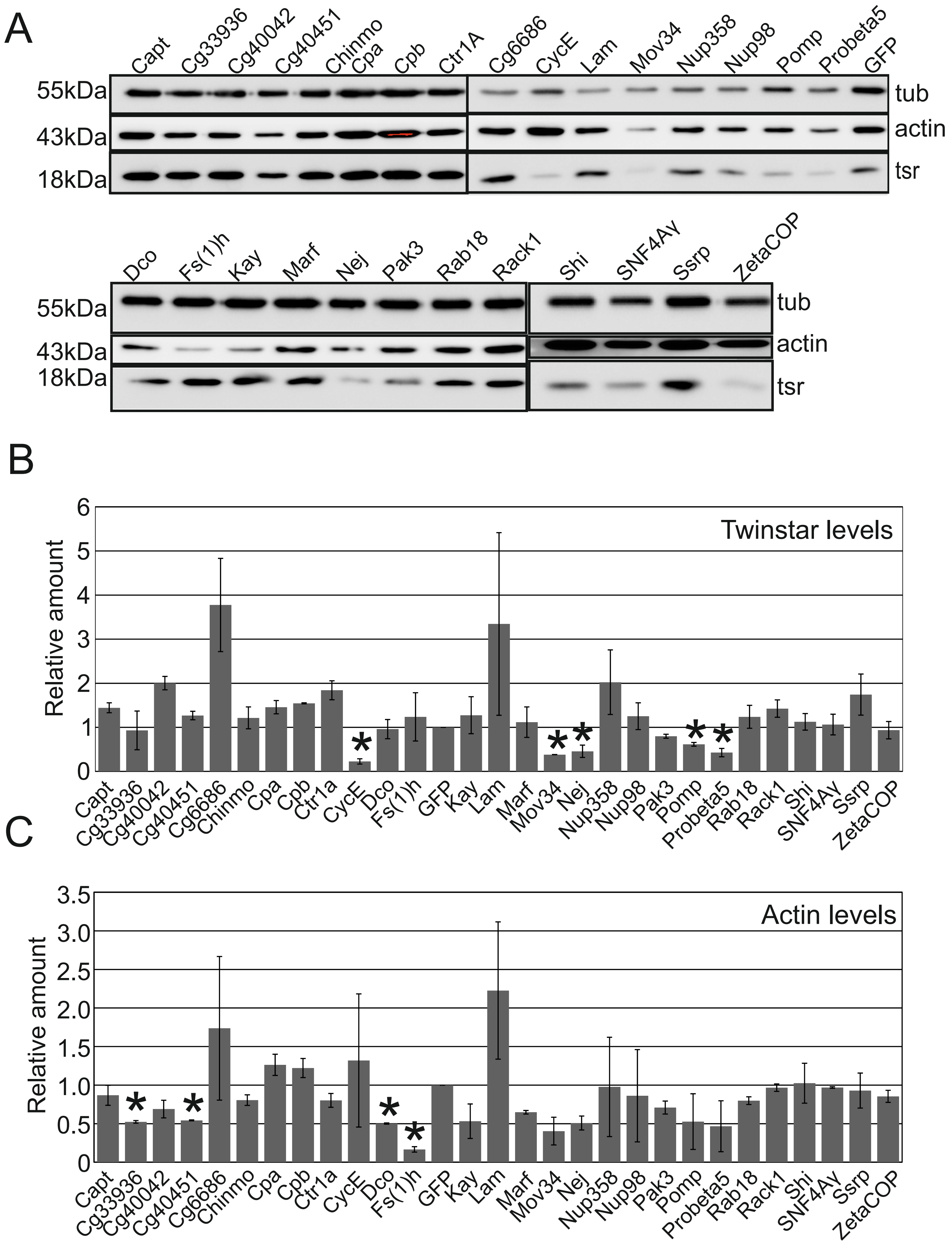

Supplemental material Figure S2

Actin and tsr levels upon depletion of the hits from the screen.

(A) Representative Western blot from whole cell lysates treated with indicated dsRNAs. GFP dsRNA was included as non-targeting control. Tubulin was detected as a sample loading control. Quantification of the levels of twinstar (B) and actin (C) from blots depicted in A. Western blot bands were quantified using NIH ImageJ and the values for actin and Tsr were normalized to the respective values of tubulin. Data is normalized to GFP, and is mean of two independent experiments, with error bars SD and statistical significance (*) with Students t-test ($p < 0.05$).

Supplementary Figure S3

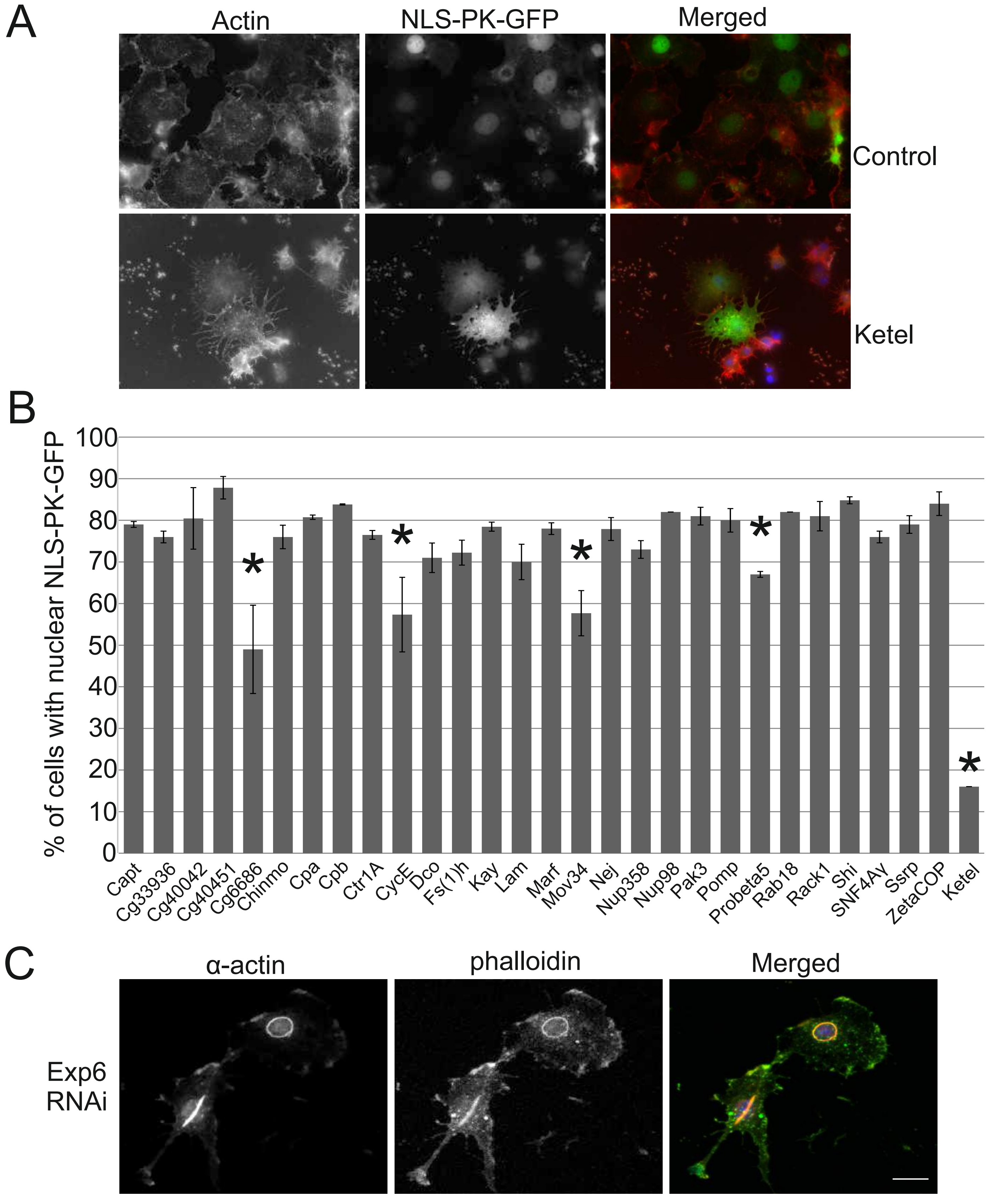

(A) Representative fluorescent microscopy images of S2R+ cells expressing NLS-pyruvate kinase-GFP (NLS-PK-GFP) (green) co-stained with phalloidin (red) and DAPI (blue). Ketel/importin ß dsRNA treated cells were included as a positive control.

(B) Quantification of the percentage of cells with nuclear NLS-PK-GFP in cells treated with the indicated dsRNAs. Data is the mean of two independent experiments (50 cells each), with error bars SD, statistical significance (*) with Students t-test ($p< 0.05$).

(C) Confocal microscopy images of Exportin 6 depleted S2R+ cells showing co-localization (merged, right panel) of actin antibody staining (left panel) and phalloidin staining (middle panel). Scale bar = 20 μm.

Supplementary Figure S4

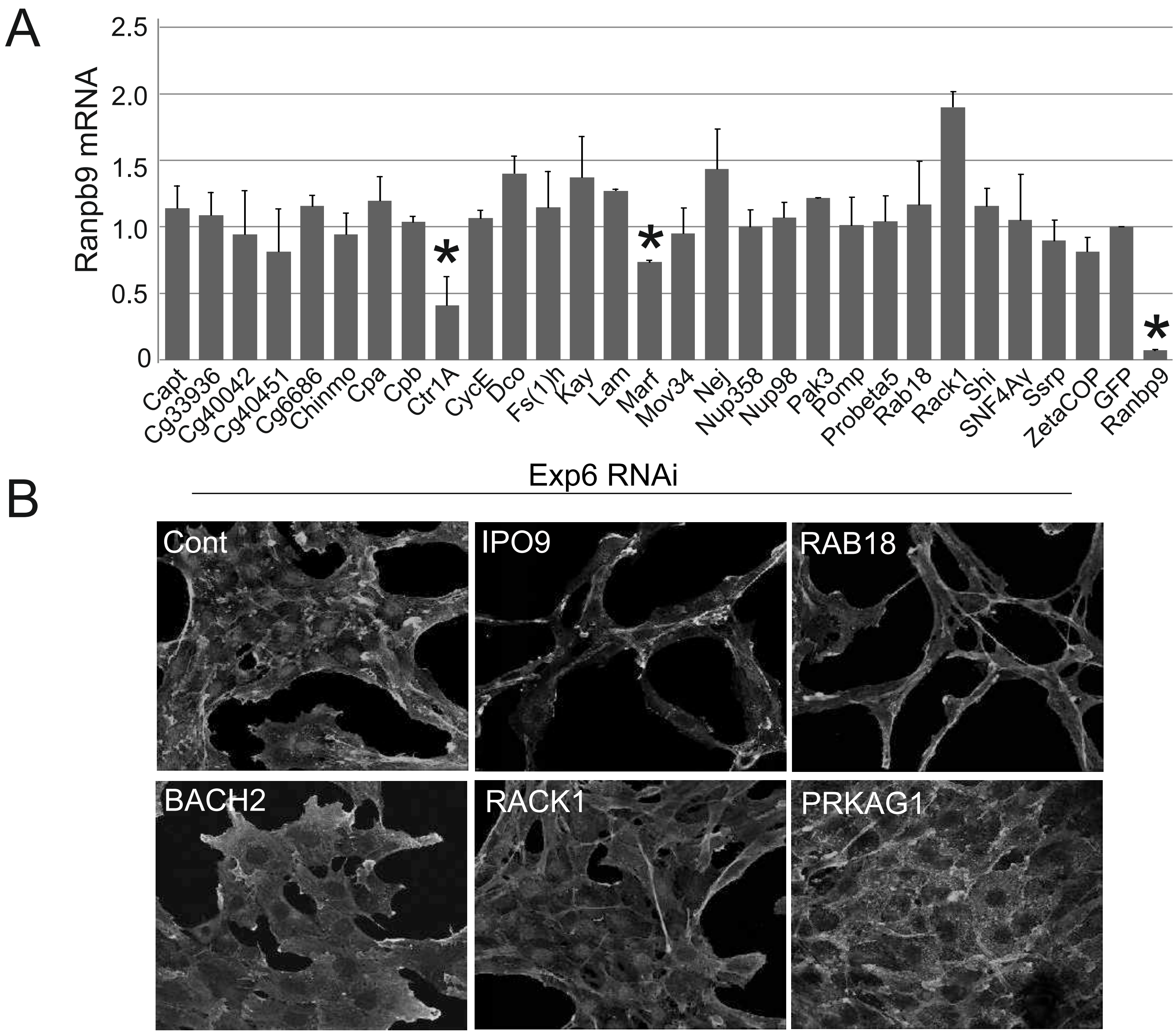

(A) RanBP9 mRNA levels in cells depleted of candidate hits. Quantification of the mRNA levels of RanBP9 in cells treated with the indicated dsRNAs. RanBP9 dsRNA was used as a control. Data is the mean of two independent experiments, with error bars SD, statistical significance (*) with Students t-test ($p < 0.05$).

(B) A subset of hits prevent exportin 6 induced nuclear accumulation of actin also in mouse cells. Representative anti-actin immunofluorescence images of NIH 3T3 cells co-transfected with siRNA against exportin 6 (EXP6) and control (Cont) or gene-specific siRNAs as indicated. Scale bar = 10 µm. Quantification in figure 7A.

Tables S1_5

Download Table S1_5
